# Hypertrophy of the feet and ankles presenting in primary hypertrophic osteoarthropathy or pachydermoperiostosis: a case report

**DOI:** 10.1186/1752-1947-6-31

**Published:** 2012-01-24

**Authors:** Rim Akrout, Samar Bendjemaa, Héla Fourati, Mariem Ezzeddine, Imene Hachicha, Soufiene Baklouti

**Affiliations:** 1 Rheumatology Department, Hedi Chaker Hospital, Sfax, Tunisia

## Abstract

**Introduction:**

Pachydermoperiostosis or primary hypertrophic osteoathropathy is a rare genetic disease with autosomal transmission. This disorder, which affects both bones and skin, is characterized by the association of dermatologic changes (pachydermia or thickening of the skin) and rheumatologic manifestations (periostosis and finger clubbing). Here, we report a new observation of pachydermoperiostosis.

**Case presentation:**

A 20-year-old North African Tunisian Caucasian man presented with hypertrophic osteoarthropathy. On a clinical examination, we found morphologic abnormalities of his face and extremities associated with skin changes. The laboratory findings were normal. A work-up disclosed no organic etiology. The final diagnosis consisted of pachydermoperiostosis syndrome.

**Conclusion:**

Pachydermoperiostosis is a rare entity that should be differentiated from secondary hypertrophic osteoarthropathy and chronic rheumatic diseases.

## Introduction

Hypertrophic osteoarthropathy (HOA) is a clinical syndrome that causes clubbing of the fingers and toes, enlargement of the extremities, and pain and swelling of the joints. Patients may have one or more of these manifestations. The syndrome can be primary, or secondary. The latter, known as hypertrophic pulmonary osteoarthropathy, is associated with pulmonary diseases such as lung cancer [1].

Pachydermoperiostosis (PDP), which is the primary idiopathic form of HOA, is characterized by clubbing of the digits of both hands and feet and enlargement of the extremities secondary to periarticular and osseous proliferation. It is a rare genetic disorder with autosomal dominant transmission. It occurs predominantly in men and has been reported in many races. Here, we report a new case of PDP seen in our Department of Rheumatology in Sfax, Tunisia.

### Case presentation

**Figure 1:**
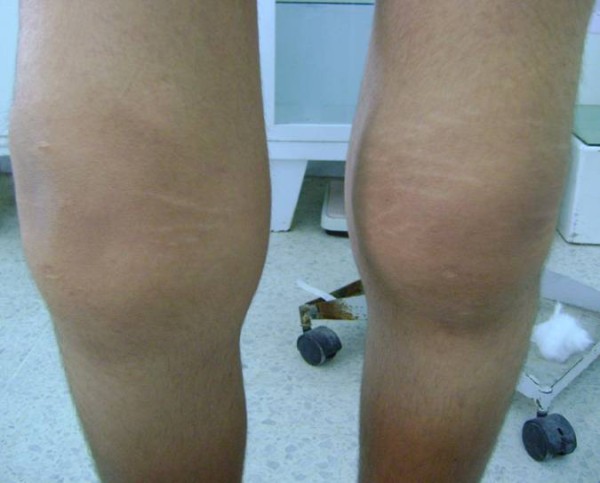
**Bilateral knee effusion**.

**Figure 2:**
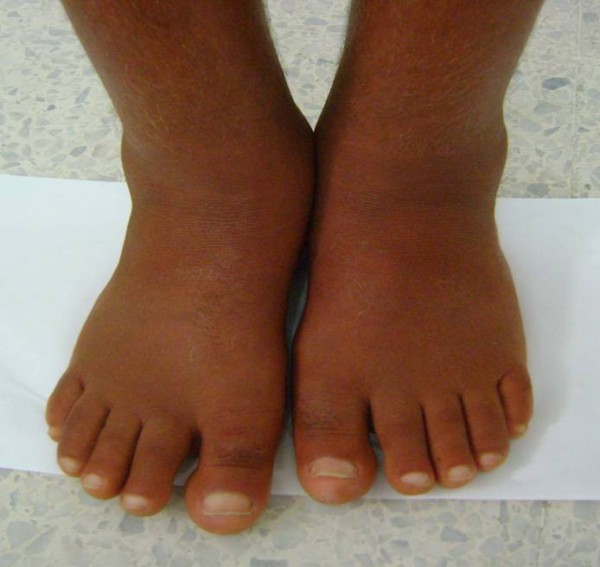
**Hypertrophy of his feet and ankles with edema**.

**Figure 3:**
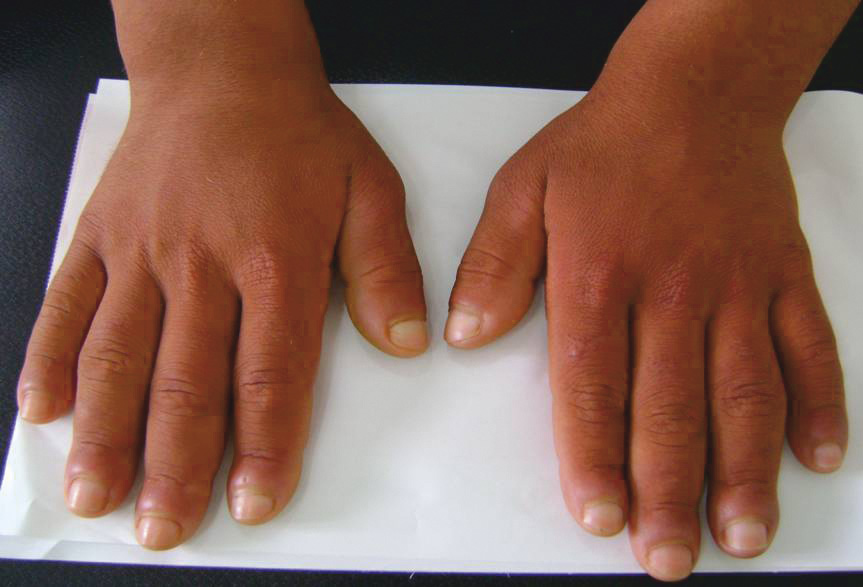
**Enlargement of his hands and deformity of his fingernails (clubbing)**.

**Figure 4:**
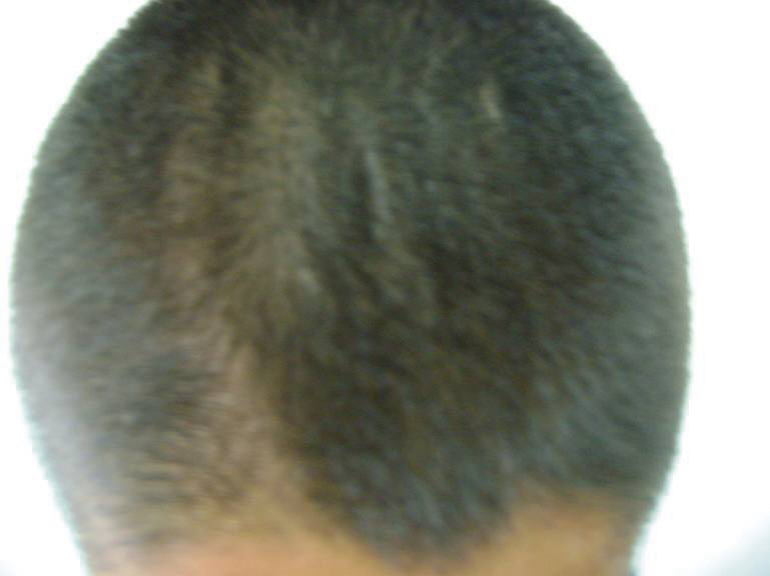
**Cutis verticis gyrata**.

**Figure 5:**
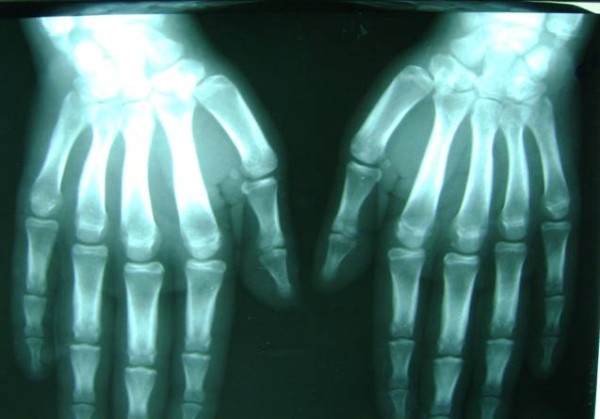
**X-ray of both hands showing periostosis**.

**Figure 6:**
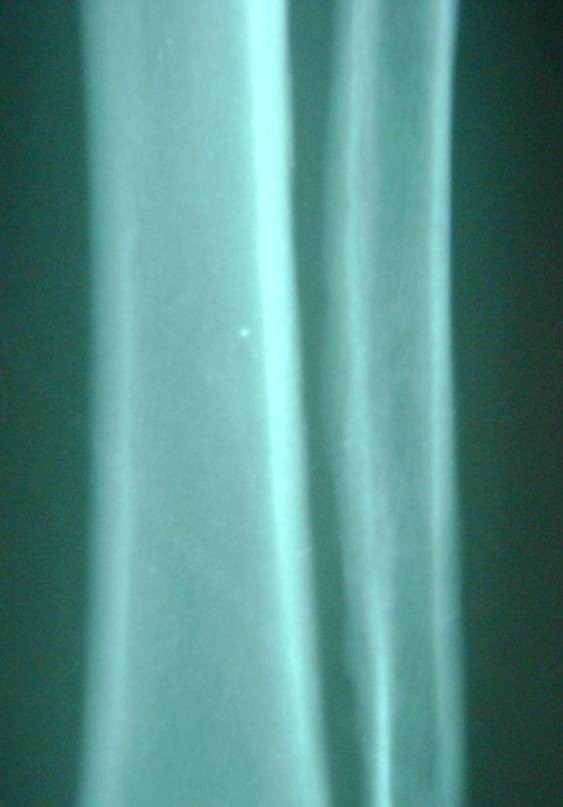
**X-ray of both legs showing periosteal reaction**.

A 20-year-old North African Tunisian Caucasian man, whose parents are first-degree cousins, presented to our department six months previously with HOA. His medical history began at the age of 10 years when he started complaining of occasional arthralgia in multiple joints after strenuous work as a farmer. He also presented with profuse sweating of his palms and soles. He noticed a progressive enlargement of his hands and feet associated with growth retardation. A physical examination revealed pachydermia with a thickening of his forehead folds, and effusion at his knees and ankles (Figures 1 and 2). Our patient denied any pain and there was no sign of local inflammation in the affected joints. There was evident clubbing of all his fingers. Others abnormalities were observed, which included enlargement of his extremities (hands and feet; Figure 3), palmoplantar hyperhidrosis and cutis verticis gyrata (Figure 4). An examination of his chest and abdomen was unremarkable. Laboratory analyses showed moderate anemia with his hemoglobin level at 11.1 g/dL, a normal erythrocyte sedimentation rate and C-reactive protein, mild polyclonal hypergammaglobulinemia at 15.5 g/L and hypocholesterolemia at 2.7 mmol/L. His liver, kidneys and all hormonal function tests were normal. An X-ray of his bones showed irregular periosteal hypertrophy with bone formation affecting his long bones, metacarpals and phalanges bilaterally (Figures 5 and 6). Scintigraphy of his bone showed a symmetrical setting of the radiotracer throughout his skeleton with a clear visualization of his entire axial and peripheral skeleton. A chest radiograph, abdominal ultrasound, echocardiograph and stomach fibroscopy were all normal.

## Discussion

HOA is divided into primary and secondary forms. PDP, the primary form, accounts for 3% to 5% of all cases of HOA [2]. Secondary HOA, also called pulmonary HOA, is associated with underlying cardiopulmonary diseases and malignancies. PDP was first reported in 1868 and it was then thought to be an example of acromegaly. The first to recognize PDP as a distinct entity from acromegaly or pulmonary HOA were Solente and Gole in 1935 (cited in [3]). The clinical manifestations are variable. Some affected patients demonstrate the complete syndrome (pachydermia, periostitis and clubbing), the incomplete form (with evidence of bone abnormalities but lacking pachydermia) or the mild form (pachydermia with minimal or absent periostitis) [3]. The diagnosis of PDP is based on the presence of at least two of the four criteria set by Borochowitz which are a history of familial transmission; pachyderma; digital clubbing; and skeletal manifestations, such as pain or signs of radiographic periostitis (cited in [4]).

Our patient had the complete form of PDP, since he had hyperostosis, finger clubbing and pachydermia. The normal results for his biological and hormonal tests are also an important argument for the condition. Most patients with idiopathic HOA have normal development until adolescence, when skin thickening and joint deformities began to occur. These changes progress for many years, then usually stabilize [5]. The disease occurs predominantly in men (sex ratio: nine to one) and is considered to be familial (25% to 40% of cases) [6]. Our patient's parents are cousins, but no other family cases were reported. Clinically, it is characterized by digital clubbing (89% of cases), pachydermia: thickening and wrinkling of facial features including the forehead and the nasolabial folds, with profound hypertrophy of the eyelids (30% to 40% of cases) and cutis verticis gyrata (24% of cases) [6]. The combination of thickened skin and bony enlargement can result in great thickening of the extremities, which is the most striking physical finding [5]. Seborrhea is noted in more than 90% of cases, with, sometimes, occurrence of acne lesions or folliculitis [6]. Hyperhydrosis is also frequent (44%) particularly in the hands and the feet and sometimes in the major folds [6]. Pubic and facial hair is almost always rare [6]. Our patient presented with all these characteristics.

Rheumatologic signs include joint effusion (41% of cases), often affecting the knees, with excess synovial joint fluid [6]. Polyarthritis can occur in 20% to 40% of cases and is often symmetrical [6]. The articular surfaces are spared, but intermittent swelling of the joints is common; they often cause moderate pain but they also can be asymptomatic, as in our case [5].

The bony changes consist of symmetric, irregular periosteal hypertrophy with new bone formation. These changes are most severe in the extremities and can involve any bone, although the skull and the vertebral column are rarely affected [5]. Radiographs revealed diffuse periostosis along the length of bones, including epiphyses, in 80% to 97% of cases, and often with irregular contours. The importance of periosteal apposition increases with disease duration. Acro-osteolysis has also been reported in 78% of cases [6]. Biologically, there is no inflammation. Hypocholesterolemia and hypergammaglobulinemia are described but unexplained [6].

Most patients have only moderate discomfort from this disease and are able to lead normal lives, as did our patient. However, the main complaints of patients are often related to their appearance and to hyperhidrosis [5]. An effective treatment for PDP is currently unknown due to the lack of controlled data and current modalities are largely based on case reports. Treatment is generally based on symptomatic therapies using non steroidal anti-inflammatory drugs, corticosteroids or colchicine [3] for pain relief. Rheumatologic symptoms can also be improved by treatment with bisphosphonates, such as pamidronic acid or risedronate. Bisphosphonates inhibit osteoclastic bone resorption and therefore reduce bone remodeling and alleviate painful polyarthritis. In some cases, plastic surgery can be helpful for improving the cosmetic appearance of the face. Fortunately, our patient did not have severe joint pain or other symptoms. Our decision was to treat him with simply oral paracetamol with a regular follow-up.

## Conclusion

The diagnosis of PDP is based on the combination of digital clubbing, periostitis and pachyderma with the absence of any cardiovascular, pulmonary, liver, intestinal or mediastinal diseases. It is a rare entity that should be known and differentiated from secondary HOA and chronic inflammatory rheumatic diseases.

## Consent

Written informed consent was obtained from the patient for publication of this case report and any accompanying images. A copy of the written consent is available for review by the Editor-in-Chief of this journal.

## Competing interests

The authors declare that they have no competing interests.

## Authors' contributions

All the authors of this article participated in the clinical work-up, the medical photography, the literature search and the writing of the manuscript. All authors read and approved the final manuscript.
